# Multicentric occurrence of multiple papillary thyroid carcinomas –HUMARA and *BRAF* mutation analysis

**DOI:** 10.1002/cam4.466

**Published:** 2015-04-17

**Authors:** Tadao Nakazawa, Tetsuo Kondo, Ippei Tahara, Kazunari Kasai, Tomohiro Inoue, Naoki Oishi, Kunio Mochizuki, Takeo Kubota, Ryohei Katoh

**Affiliations:** 1 Department of Pathology, Interdisciplinary Graduate School of Medicine and Engineering, University of YamanashiYamanashi, Japan; 2 Department of Epigenetic Medicine, Interdisciplinary Graduate School of Medicine and Engineering, University of YamanashiYamanashi, Japan

**Keywords:** *BRAF* mutation, clonal analysis, HUMARA, intraglandular metastasis, multicentric occurrence, multiple papillary thyroid carcinoma, X-chromosome inactivation pattern

## Abstract

Papillary thyroid carcinomas (PTCs) occasionally form multiple tumor foci in different sites of the same thyroid gland. However, it is controversial whether discrete nodules of PTC arise independently (multicentric occurrence) or are seeded from a single tumor via lymphatic channels (intraglandular metastasis). In order to determine the clonal origin of multiple PTCs, we examined X-chromosome inactivation patterns using a human androgen receptor gene-based assay (HUMARA) and the *BRAF* mutation using allele-specific PCR (AS-PCR) in 32 microdissected cancerous tissues from 14 Japanese women with multifocal PTC. All tumor foci were greater than 3 mm in size and met the criteria for microscopic classical PTC. Samples from 13 of the 14 patients were informative based on HUMARA. Tumor foci from two cases (15.4%) displayed a discordant X-chromosome inactivation pattern. Foci from the other 11 cases (84.6%) showed a concordant inactivation pattern of the X-chromosome. AS-PCR indicated that *BRAF* mutational status between the tumor foci was discordant in three (25%) and concordant in nine (75%) of 12 available cases. When the results of these two molecular analyses were combined, 28.6% of the cases were discordant in X-chromosome inactivation pattern and/or *BRAF* mutation, suggesting multicentric origin. Some of the remaining concordant cases also may be of multicentric origin. These results support a hypothesis that multicentric occurrence in multiple PTCs may be common, possibly greater than 30%. Although the exact mechanism of multicentric occurrence is still unclear, our findings contribute to the understanding the histogenesis of papillary thyroid carcinoma.

## Introduction

Papillary thyroid carcinoma (PTC) is the most common thyroid carcinoma, accounting for approximately 90% of all thyroid malignancies 1–3. They can form multiple, discrete tumor nodules in the thyroid gland 4–6, and although minute microscopic disseminations of PTC are common, multiple PTC foci that are large and visible macroscopically are relatively rare. When there are multiple foci, it is difficult for pathologists to differentiate the true primary tumor.

There is a consensus that microscopic, metastatic foci of PTC within the thyroid gland and metastases to the regional lymph nodes are generated through lymph vessels in the thyroid gland. This biological characteristic of PTCs can explain the multiplicity of PTCs, namely intraglandular metastasis. On the other hand, several reports have demonstrated different subtypes of *RET/PTC* and different *BRAF* mutational statuses in individual PTC foci among patients with multifocal PTCs 7–10. These divergent genetic profiles suggest that each tumor is derived from an independent clonal origin.

To date, there have been five reports that used Human androgen receptor gene-based assay (HUMARA, Table1) to the clonal origins of individual PTC foci 11–15; they provide conflicting evidence. McCarthy et al. showed concordance between foci of a nonrandom X-chromosome inactivation pattern in all informative cases 11. A more recent study showed a high frequency of the same inactivation pattern (nine of 11 cases), which suggests that individual tumors arise from a single clone and that intraglandular metastasis may play an important role in the formation of multifocal PTCs 12. In contrast, Moniz et al. demonstrated that a distinct allele of the X-chromosome was inactivated in different tumor foci in three out of eight cases with multifocal PTC, suggesting independent clonal origins of these foci 13. Similarly, Shattuck et al. reported on a discordant X-chromosome inactivation pattern between individual tumor foci in half of the cases (five out of 10) examined 14. In addition, a high frequency of discordant X-chromosome inactivation patterns was detected in contralateral PTCs 15.

**Table 1:** Reported concordance of X-chromosome inactivation patterns in multifocal papillary thyroid carcinoma by HUMARA

Authors [ref. no.]	No. of informative cases (*n*)	Concordant cases	Discordant cases
Moniz et al. 13	8	5 (62.5%)	3 (37.5%)
Shattuck et al. 14	10	5 (50%)	5 (50%)
McCarthy et al. 11	21	21 (100%)	0
Wang et al. 12	11	9 (81.8%)	2 (18.2%)
Kuhn et al. 15	8	3 (37.5%)	5 (62.5%)
Current study	13	11 (84.6%)	2 (15.4%)

Although many investigators in the field of thyroid pathology are interested in the pathogenesis of multifocal PTCs, the principal mechanism responsible for multifocal PTCs must still be determined. In the current study, we examined X-chromosome inactivation patterns and *BRAF* mutations of multiple PTCs using HUMARA and AS-PCR, respectively, to determine the clonal origins of individual PTC nodules.

## Materials and Methods

### Patients and tissue preparation

We obtained specimens from 32 surgically resected PTCs from 14 patients on file at Yamanashi University Hospital. All patients were female with no history of irradiation or clinical presentation of Hashimoto’s thyroiditis. Table1 shows the clinicopathological features of the patients. Preoperatively, all patients presented for “multiple thyroid tumors” seen on ultrasonography. Each patient had two or three PTC nodules measuring at least 4 mm. The pathological diagnoses were *The World Health Organization Classification of Tumour, Pathology and Genetics of Tumours of the Endocrine Organs* 16. Three pathologists (T. N., T. K., and R. K.) reviewed the hematoxylin and eosin (H&E)-stained sections and agreed in all cases. All tumor nodules were the classical type of PTC with neither distinct variants, nor anaplastic or poorly differentiated components. All patients gave their informed consent.

### Microdissection and DNA extraction

**Figure 1:**
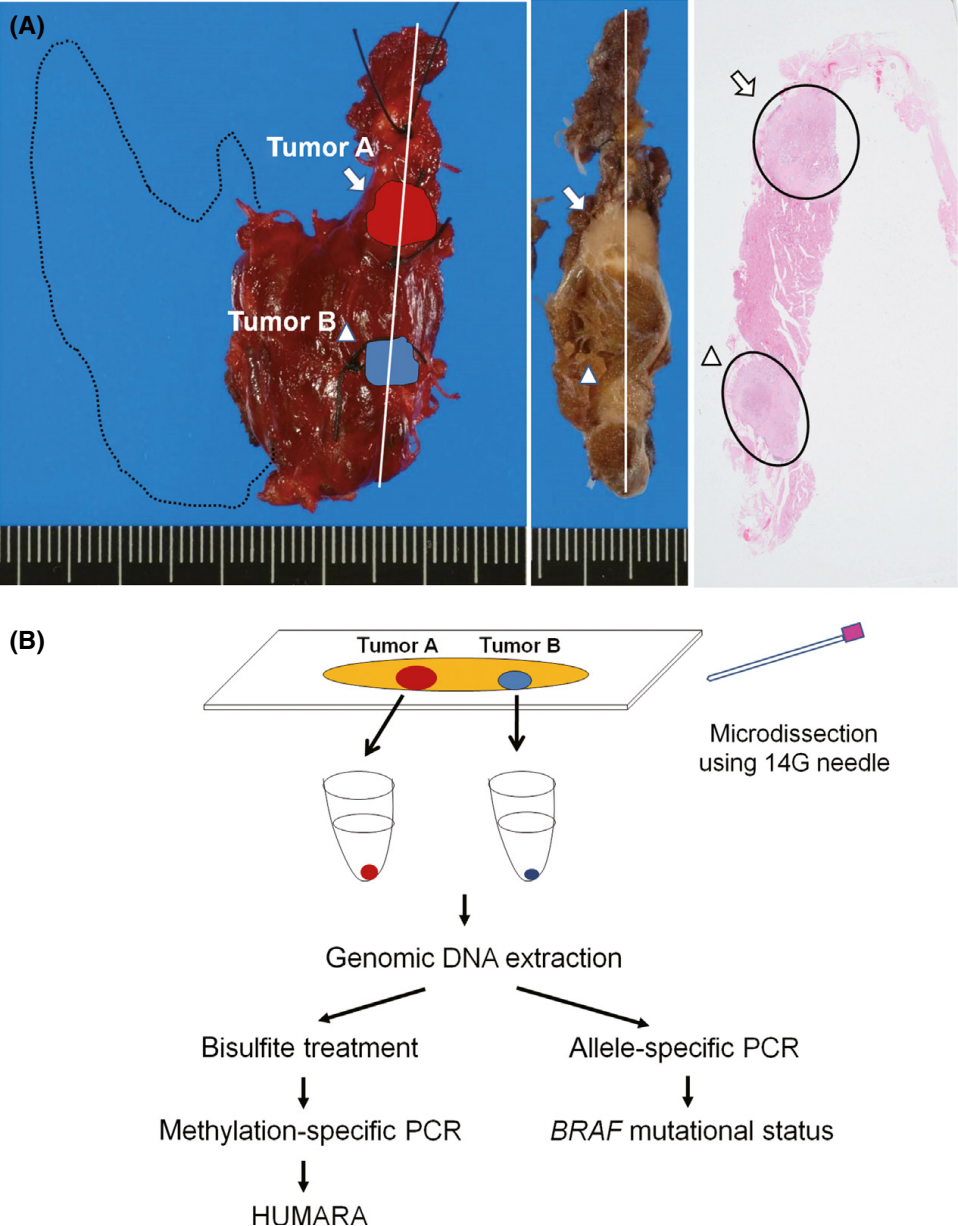
Example of microdissection of multiple papillary thyroid carcinomas (PTCs) for clonal analysis and a schema of human androgen receptor gene-based assay (HUMARA) and allele-specific PCR (AS-PCR). (A) Section of thyroid gland from a representative case of multifocal PTC (case 1). The far-left picture shows a right-hemithyroidectomy specimen. Noncontinuous tumor foci are present (tumor A, arrow; tumor B, arrow head) on the cut surface (middle picture) and the corresponding H&E-stained section (far-right picture). (B) Genomic DNA was independently extracted from tumor A and tumor B by microdissection using a 14G needle, followed by methylation-specific PCR and then HUMARA. The DNA was also analyzed for *BRAF* mutation using AS-PCR.

Figure1 shows the protocol for tumor microdissection followed by DNA extraction. A 4-*μ*m-thick section was cut from routinely processed, formalin-fixed and paraffin-embedded blocks and stained with H&E for microscopic examination. To extract the tumor DNA, four serial, unstained 10-*μ*m-thick sections were cut and mounted on glass slides. To maintain orientation, tumor margins were marked on the corresponding H&E-stained slide. By comparing stained and unstained sections, tumor tissue could be microdissected from the unstained sections using a disposable 14G syringe needle. We deparaffinized the collected pieces of tumor tissue with xylene, rehydrated them graded ethanol, and then extracted the genomic DNA using a phenol–chloroform and alcohol precipitation kit (RecoverAll Total Nucleic Acid Isolation Kit; Ambion, Austin, TX) according to the manufacturer’s instructions.

### Human androgen receptor gene-based assay

Prior to HUMARA, isolated DNA was treated with sodium bisulfite as follows: DNA (0.2–1 *μ*g) was incubated at 55°C overnight with hydroquinone and sodium bisulfite (Sigma, St. Louis, MO) and then purified using the Wizard DNA clean-up system (Promega, Madison, WI). After ethanol precipitation, the treated DNA was resuspended in 50 *μ*L TE (10 mmol/L TRIS, 1 mmol/L EDTA, pH 8.0).

Methylation-specific PCR was carried out as previously described 17,18. Briefly, one primer was labeled with the fluorescent dye Cy5 (DyeAmidite-667; Pharmacia, Kalamazoo, MI). The polymerase was activated at 95°C for 10 min. DNA was amplified in a thermal cycler for 35 cycles at 94°C for 30 sec, 58°C for 30 sec, and 72°C for 30 sec, followed by a final extension at 72°C for 10 min. We separated the amplified PCR products by high-resolution fluorescence electrophoresis, and detected the products with an ABI PRISM 377 DNA automatic sequencer (Applied Biosystems, Foster City, CA). GeneScan 3.1 software visualized individual gel lanes as electropherograms for each detected fluorescent peak.

**Figure 2:**
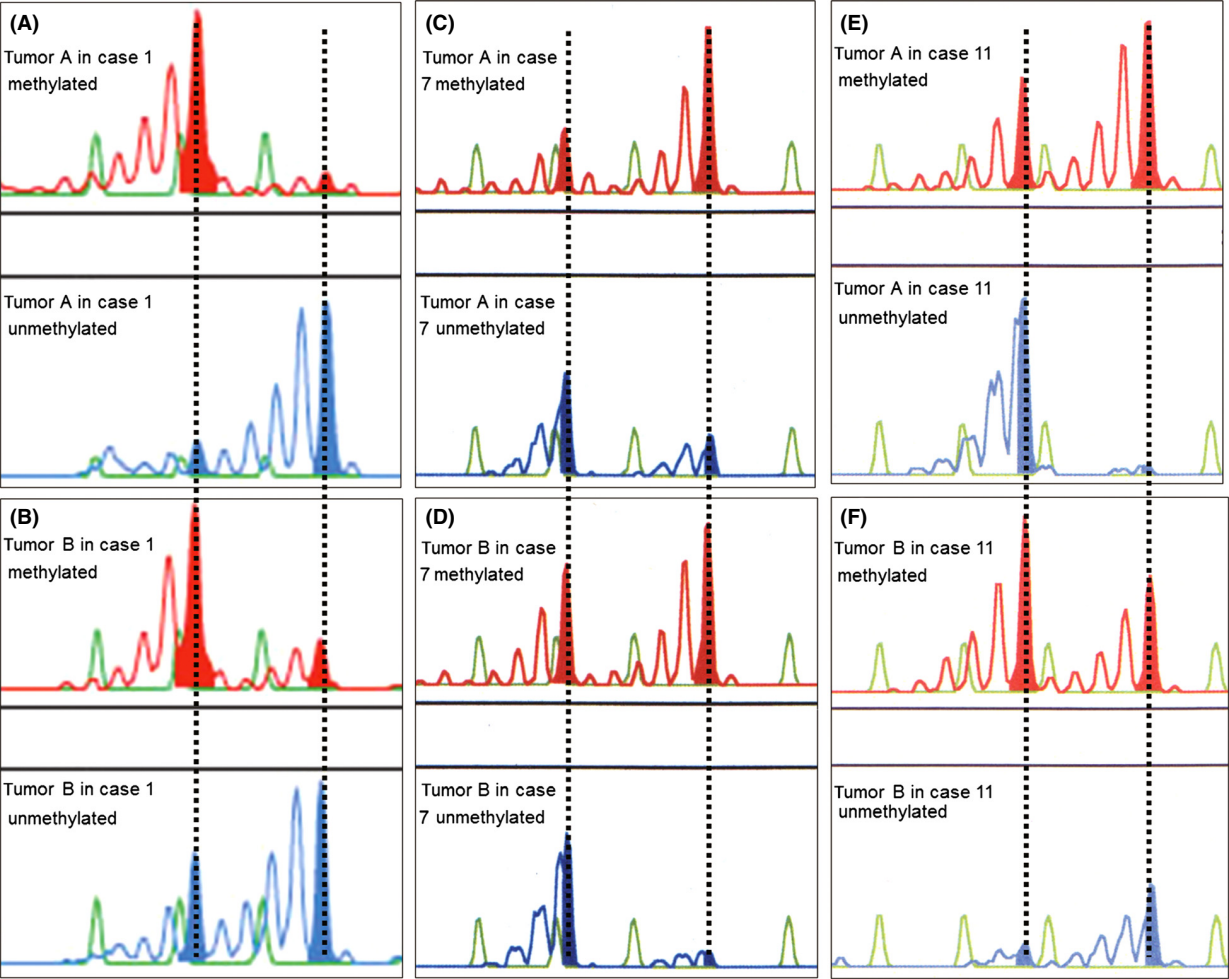
(A-F) Representative results of X-chromosome inactivation patterns by HUMARA. Red- and blue-colored bars represent polymerase chain reaction (PCR) products for methylated- and unmethylated-alleles, respectively. (A) In case 1, the short allele of the androgen receptor is inactivated in tumor A (pattern S). (B) In tumor B, HUMARA shows that the short allele of X-chromosome is inactivated (pattern S). (C) In case 7, the long allele of the androgen receptor is inactivated in tumor A (pattern L). (D) HUMARA showed the same X-chromosome inactivation pattern in tumor B. (E) In case 11, the long allele of the androgen receptor is inactivated in tumor A (pattern L), (F) whereas the short allele is inactivated in tumor B (pattern S).

In informative cases, there were two fluorescence peaks in the PCRs targeted toward both methylated and unmethylated androgen receptor (AR) genes due to contamination by nonneoplastic cells in the microdissected tissues. Therefore, we used the predominant fluorescent peak to determine the inactivation pattern of the tumor foci. In detail, we determined inactivation of the short allele (pattern S) based on the following conditions: (1) dominant methylation pattern at the fluorescent peak area of the short allele and (2) dominant nonmethylation pattern at the long allele (Figs.2A, B, and F). Adverse conditions were assessed as inactivation of the long allele (pattern L) (Fig.2C, D, and E).

### Allele-specific polymerase chain reaction (AS-PCR)

We amplified 5 *μ*L of extracted DNA in a total volume of 50 *μ*L using 500 nmol/L of primer and the HotStarTaq Mix Kit (Qiagen, Tokyo, Japan). The primer sequences for *BRAF* V600E were 5′-GGT GAT TTT GGT CTA GCT ACA TA-3′ and 5′-GGC CAA AAA TTT AAT CAG TGG A-3′; amplicon size, 126 base pairs. Primers for wild-type *BRAF* acted as an internal control, and the sequences were as described previously 19. The amplifications occurred as follows: 15 min at 94°C, followed by 40 cycles of 95°C for 15 sec and 72°C for 30 min, with a final extension at 72°C for 10 min. Electrophoresis of the polymerase chain reaction products occurred in a 3% agarose gel with ethidium bromide and then visualized under ultraviolet light. The thyroid-cancer-derived cell lines KTC-1 and WRO acted as positive and negative controls, respectively.

### Statistical analysis

The probability (*P*) of cases sharing the same clonal origin and the standard error (SE) of the *P* value were calculated according to the statistical inference out of binomial distribution theory. We considered cases with three PTC foci as “concordant,” only when all three foci had the same X-inactivation pattern, which leads to underestimation of the *P* value.

## Results

### Clinicopathological findings

Table2 shows the clinicopathological features of our 14 cases with multiple PTCs. The patients’ ages ranged from 35 to 76 years (mean 53.5 years). The PTC nodules measured from 4 to 18 mm in size (mean 9.2 mm). Seven cases had PTC foci located in both lobes, and seven cases had only unilateral lobe involvement. Nine of 12 cases showed PTC lymph node metastasis. The duration of follow-up was from 2 to 136 months (mean 43.1 months). We found additional microscopic foci of PTC in five cases, six cases had focal lymphocytic thyroiditis, and one case had Graves’ disease (case 10). Of the 13 cases, 12 patients are alive without evidence of recurrence or distant metastases, and one patient died of lung cancer dissemination.

**Table 2:** Clinicopathological features of 14 patients with multiple papillary thyroid carcinoma

Case	Age	Tumor	Tumor size (mm)	Location	Extrathyroidal extension	Lymph node metastasis	Additional microscopic tumor foci	Focal thyroiditis	Follow-up (mo)	Prognosis
1	65	A	8	Left	+	NA	-	-	60	AWOD
		B	4	Left	+					
2	49	A	9	Left	-	+	+	-	55	AWOD
		B	6	Left	+					
		C	4	Left	-					
3	53	A	18	Right	+	+	-	-	52	AWOD
		B	13	Left	−					
		C	10	Left	+					
4	35	A	15	Right	+	+	-	+	82	AWOD
		B	10	Right	+					
5	69	A	8	Right	+	+	-	-	79	AWOD
		B	4	Left	-					
6	48	A	16	Right	+	+	-	+	136	AWOD
		B	5	Right	-					
7	53	A	17	Left	-	NA	+	-	48	Dead for lung cancer
		B	9	Right	-					
8	43	A	10	Left	+	-	-	-	37	AWOD
		B	8	Right	-					
9	40	A	14	Right	+	+	+	+	31	AWOD
		B	7	Right	-					
		C	9	Left	-					
10	57	A	7	Left	+	-	-	-	19	AWOD
		B	6	Left	-					
11	76	A	12	Left	+	+	-	-	15	AWOD
		B	8	Left	+					
12	50	A	6	Right	+	-	+	+	3	AWOD
		B	6	Left	+					
13	57	A	12	Left	-	+	+	+	2	AWOD
		B	10	Right	+					
		C	7	Right	-					
14	54	A	10	Right	-	+	-	+	16	AWOD
		B	6	Left	-					

AWOD, alive without disease; NA, not available.

### X-chromosome inactivation patterns

HUMARA indicated that 13 of 14 cases were informative (92.9%). Figure2 shows representative cases of concordant and discordant X-chromosome inactivation patterns as assessed by HUMARA. In case 1, the short allele of the AR was inactivated in both tumor A and tumor B (pattern S) (Figs.2A and B). In case 7, the long allele of the AR was inactivated in both tumor A and tumor B (pattern L) (Figs.2C and D). Discrete PTC foci displayed the concordant X-chromosome inactivation pattern in these cases.

In case 11, the short allele was inactivated in tumor A (pattern S) (Fig.2E), while the long allele was inactivated in tumor B (pattern L) (Fig.2F). HUMARA revealed that the X-chromosome inactivation pattern of tumor A was different from tumor B’s pattern, leading to the discordant case.

Table2 shows a summary of the X-chromosome inactivation patterns as assessed by HUMARA. Tumor foci from two cases (15.4%) (cases 3 and 11) were discordant in their X-chromosome inactivation patterns. In contrast, tumors from the other 11 cases (84.6%) demonstrated X-chromosome inactivation patterns.

Statistically, the expected probability (*P*) value for multiple tumor foci sharing the same progenitor cell was 69.2% among the 13 informative cases with multiple PTCs, and the SE of probability value was 12.8%. Therefore, the 95% confidence interval (CI) was from 56.4% to 94.3%.

### *BRAF V600E* mutational status

**Figure 3:**
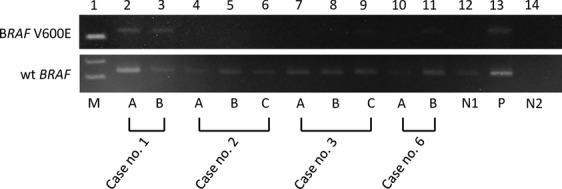
Analysis of *BRAF* mutation in multifocal papillary thyroid carcinomas (PTCs). Representative results of *BRF* V600E detected by allele-specific polymerase chain reaction in multifocal papillary thyroid carcinomas (PTCs) (Lanes 2–11 in the upper figure). The lower figure indicates results for exon 15 of wild-type *BRAF* as a quality control of extracted DNA. Lane 1 (M) is a marker. Positive bands for *BRAF* V600E are shown in Lanes 2, 3, 9, and 11 corresponding to tumor A and B from case 1, tumor C from case 3, and tumor B from case 6, respectively. Lane 13 (P) is a positive control for *BRAF* V600E (KTC-1), while lane 12 (P) as negative control (WRO). Lane 14 (N2) represents a negative control (no template).

Figure3 shows representative results of *BRAF* V600E mutations. We could compare the *BRAF* V600E status of different tumor foci using AS-PCR in 12 of our 14 cases. In three of these 12 cases (25%), discrete PTC foci showed a discordant *BRAF* mutational status: in case 3, tumor A had the *BRAF* mutation, while tumors B and C did not; in cases 6 and 12, tumor A lacked the *BRAF* mutation, whereas tumor B displayed it. *BRAF* mutational status was concordant between different tumor foci in nine of the cases 12 (75%); all tumors were positive for the mutation in four cases, and all were negative in five cases.

### Combined results of X-chromosome inactivation pattern and *BRAF* mutational status

Table3 shows the HUMARA and/or AS-PCR results of the tumor foci from all 14 patients. In case 3, there was discordant in both the *BRAF* mutational status and the X-chromosome inactivation pattern. In case 11, *BRAF* mutational status was concordant, although the tumors displayed different X-chromosome inactivation patterns by HUMARA. Of note, in cases 6 and 12, *BRAF* mutational status differed between tumors, whereas the X-chromosome inactivation pattern was discordant. In summary, we detected a discordant X-chromosome inactivation pattern and/or *BRAF* mutational status in 4 of 14 cases (cases 3, 6, 11, and 12) (28.6%).

**Table 3:** X-chromosome inactivation patterns by HUMARA and *BRAF* mutational status in 14 multiple papillary thyroid carcinomas

Case	Tumor	Methylated (inactivated) X-chromosome allele	Concordance by HUMARA	BRAF V600E	Concordance by BRAF mutation
1	A	S	Concordant	+	Concordant
	B	S		+	
2	A	L	Concordant	-	Concordant
	B	L		-	
	C	L		-	
3	A	S	Discordant	-	Discordant
	B	L		-	
	C	L		+	
4	A	S	Concordant	NA	NA
	B	S		-	
5	A	L	Concordant	NA	NA
	B	L		NA	
6	A	S	Concordant	-	Discordant
	B	S		+	
7	A	L	Concordant	+	Concordant
	B	L		+	
8	A	S	Concordant	+	Concordant
	B	S		+	
9	A	S	Concordant	-	Concordant
	B	S		-	
	C	S		-	
10	A	L	Concordant	-	Concordant
	B	L		-	
11	A	L	Discordant	-	Concordant
	B	S		-	
12	A	L	Concordant	-	Discordant
	B	L		+	
13	A	L	Concordant	+	Concordant
	B	L		+	
	C	L		+	
14	A	Not informative	NA	-	Concordant
	B			-	

L, long allele; S, short allele; NA, not available.

### Correlation with clinicopathological parameters

We found the same rate of discordant X-chromosome inactivation pattern and/or *BRAF* mutational status (28.6%, 2/7 cases) in tumors whether there was bilateral or unilateral lobe involvement (Tables2 and 3). The proportions of discordant X-chromosome inactivation pattern and/or *BRAF* mutational status were 20% (1/5 cases) when there were additional microscopic foci and 30% (3/9 cases) when there were no additional microscopic foci. The X-chromosome inactivation pattern and/or *BRAF* status had the same rate of discordance in patients with lymph node metastasis (3/9, 33.3%) and in patients without metastasis (1/3, 33.3%). Furthermore, the rate of discordant X-chromosome inactivation pattern and/or *BRAF* status was the same between patients with focal lymphocytic thyroiditis (33.3%, 2/6) and without focal lymphocytic thyroiditis (25%, 2/8). No patients experienced recurrence or distant metastasis of the PTC, regardless of the X-chromosome inactivation pattern and *BRAF* mutational status.

## Discussion

Thyroidectomy specimens may contain multifocal nodules of PTC. Both multicentric occurrence and intrathyroidal metastasis can explain the pathogenesis of multifocal PTCs. However, there is still controversy over correct explanation, and interest in this issue has increased among thyroid researchers. Clarifying the mechanism may be important in treatment decisions and determining prognoses for patients with multifocal PTC. Therefore, we tried to determine the pathogenesis of multifocal PTC by elucidating the clonal origin of individual PTC foci. We examined X-chromosome inactivation patterns and the *BRAF* mutational status of 32 PTCs from 14 patients using HUMARA and AS-PCR, respectively. None of the tumors examined in this study was less than 4 mm in size, and we excluded all microscopic PTC foci.

Table1 shows the remarkably varied concordance rates of X-chromosome inactivation pattern from different studies as determined with HUMARA. This can be attributed to two reasons. First, during the manual microdissection of the tumors, some DNA from nonneoplastic cells may have been included 12,15. In general, we could discern a considerable amount of stromal cells in the PTCs. A tumor sample that was highly contaminated with DNA from nonneoplastic cells might not demonstrate a clearly skewed X-chromosome inactivation pattern. Laser capture microdissection would enable us to purify DNAs from neoplastic cells. Another reason for the variability that distinct variants of PTC were included in the analyzed PTCs 12,15. We selected discrete PTC foci that were all microscopically classical PTC and could not be distinguished.

Correlation between clonality and clinical outcome is still a matter of debate in thyroid carcinomas. In the current study, four of the 14 cases (28.6%) with multifocal PTCs had a discordant X-chromosome inactivation pattern and/or *BRAF* mutational status, as determined using a combination of two assay methods. This result indicated that at least 28.6% of multifocal PTCs were from different clones and arose independently. None of the clinical parameters in our cases correlated with the clonality of discrete PTC foci or indicated patients’ prognoses. However, we examined only a small number of cases multiple PTCs in this study. Furthermore, noninformative results were included. Additional studies using a larger series could clarify the actual impact of clonality on clinical outcomes.

*BRAF* and *RAS* point mutations and *RET* gene rearrangement (*RET/PTC*) are widely known as representative genetic alterations in PTCs 19–21. In multifocal PTCs, previous studies provided evidence that *BRAF* mutational status or subtypes of *RET*/*PTC* differed among tumor foci 7–10. Although these genetic diversities occasionally emerge in clonal evolution during tumor development 22, it is conceivable that individual tumors are derived from independent progenitor cells. Currently, *BRAF* mutations are the most common mutations in PTCs and are detected in 40-45% of PTCs, while both *RAS* and *RET/PTC* account for 10–15% of the genetic abnormalities of PTCs 23. Moreover, only a small population of cancer cells have *RET/PTC*s*,* suggesting that *RET/PTC*s are subclonal events 24. Based on these data, we selected the *BRAF* mutation from the representative genetic alterations for clonal analysis of multifocal PTCs and compared *BRAF* mutational status between discrete PTC foci.

Taking into account that a maternal or paternal X-chromosome is inactivated by chance, a concordant X-chromosome inactivation pattern revealed by HUMARA does not necessarily reflect the same clonal origin of discrete foci. The incidence of different tumor foci sharing the same clonal origin is limited to speculation through statistical analyses. Conversely, different tumors having a discordant X-chromosome inactivation pattern would necessarily come from different progenitor cells. We can apply a similar theory the *BRAF* mutational status for the PTC foci, because *BRAF* mutations have been identified in about half of the PTCs examined 23. For these reasons, we combined HUMARA to detect X-chromosome inactivation pattern and AS-PCR to detect the *BRAF* mutation and provide a more precise proportion of multifocal PTCs with different clonal origins.

There have been three previous studies using *BRAF* mutational states in multiple PTCs for clonal analysis 8,10,12. In one study, there were three of 21 cases (15.3%) with multifocal PTCs having discordant *BRAF* mutational status 12. In a large series of Korean patients (61 patients), Park et al. detected a different *BRAF* status in at least 39.3% of multifocal PTCs 8. More recently, 26 of 60 multiple PTCs (43.3%) harbored a different *BRAF* status 10. In our study, three of 12 multiple PTCs (25%) showed a discordant status, although we examined only a small number of cases.

Epithelial cancers with multiple foci of other organs, such as multiple bladder tumors, frequently occur synchronously and/or metachronously. Molecular analyses have demonstrated that most of these tumors showed the same X-chromosome inactivation pattern when analyzed by HUMARA and the same genetic abnormalities when analyzed by other molecular approaches, such as loss of heterozygosity (LOH) and p53 mutation 25,26. These data support the possibility of intramucosal seeding/spreading that is analogous to intraglandular metastasis of multiple PTCs in the thyroid gland. Likewise, in eight of 10 cases of bilateral breast carcinoma, contralateral tumors were likely to originate from the same clone based upon microscopic resemblance as well as on a high proportion of concordant X-chromosome inactivation patterns revealed by HUMARA 27. On the other hand, most hepatocellular carcinomas (HCCs) arise independently in a lineage of the macronodules of cirrhosis 28. Patients with multicentric HCC showed significantly better overall and recurrence-free survival compared with patients with HCC showing intrahepatic metastasis (same origin) 29. However, in other organ cancers including thyroid carcinomas, it is not clear whether there is a difference in clinical outcome between patients with multicentric tumors and patients with tumor foci having the same clonal origin.

In summary, molecular analyses using HUMARA and AS-PCR showed that four of 14 patients (28.6%) with multiple PTC had discrete PTC foci with a different X-chromosome inactivation pattern and/or *BRAF* mutational statuses; nearby 30% of the multiple PTCs shared a different clonal origin. In addition, more cases with multicentric PTCs can be included in the residual cases with those having concordant X-chromosome inactivation pattern and *BRAF* mutational status. Molecular analyses using a Next Generation Sequencer will give us more precise information as to their clonality. The high frequency of multicentric PTC may indicate that a very small number of genetic events are necessary for the synchronous occurrence of each PTC focus.

## Conflict of interest

The authors declare that they have no conflict of interest.
